# Identifying genes associated with invasive disease in *S. pneumoniae* by applying a machine learning approach to whole genome sequence typing data

**DOI:** 10.1038/s41598-019-40346-7

**Published:** 2019-03-11

**Authors:** Uri Obolski, Andrea Gori, José Lourenço, Craig Thompson, Robin Thompson, Neil French, Robert S. Heyderman, Sunetra Gupta

**Affiliations:** 1 University of Oxford, Department of Zoology, Oxford, UK; 2 University College London, Division of infection and immunity, London, UK; 3 Liverpool School of Tropical Medicine, Liverpool, UK

## Abstract

*Streptococcus pneumoniae*, a normal commensal of the upper respiratory tract, is a major public health concern, responsible for substantial global morbidity and mortality due to pneumonia, meningitis and sepsis. Why some pneumococci invade the bloodstream or CSF (so-called invasive pneumococcal disease; IPD) is uncertain. In this study we identify genes associated with IPD. We transform whole genome sequence (WGS) data into a sequence typing scheme, while avoiding the caveat of using an arbitrary genome as a reference by substituting it with a constructed pangenome. We then employ a random forest machine-learning algorithm on the transformed data, and find 43 genes consistently associated with IPD across three geographically distinct WGS data sets of pneumococcal carriage isolates. Of the genes we identified as associated with IPD, we find 23 genes previously shown to be directly relevant to IPD, as well as 18 uncharacterized genes. We suggest that these uncharacterized genes identified by us are also likely to be relevant for IPD.

## Introduction

Invasive pneumococcal disease (IPD) is defined as an infection in which the bacterial pathogen *Streptococcus pneumoniae* (pneumococcus) enters a usually sterile site, such as the blood or cerebrospinal fluid^1^. Although pneumococci are usually carried asymptomatically within the human nasopharynx, IPD is often life-threatening and constitutes a major cause of mortality, disproportionally targeting children, elderly and immune-suppressed individuals^2,3^. Genetic changes facilitating the survival of pneumococci during invasion have been previously identified and described through experimental and bioinformatic methods^4–10^. The work of Hava and Cammilli, for instance, describes a set of 378 genes that are associated with attenuated virulence in mice in the model pneumococcal strain TIGR4^4^. Several other works have been successful in identifying differential expression patterns of key virulence genes of *S. pneumoniae in vitro* and *in vivo*. These works used RT-PCR on previously described virulence factors and high-throughput microarray expression profiling to identify gene expression signatures during invasion of model organisms or growth on epithelial cell lines^5,6^. DNA microarrays have also been employed in order to identify a common core genome differentiating between strains isolated from invasive disease or carriage in three pneumococcal serotypes often found in IPD (6A, 6B and 14)^8^. Although these methods did highlight features involved in the ability of pneumococci to invade a host, they were limited by either using a small sample size, focusing only on a fraction of the pneumococcal serotypes, or relying on a single reference genome to identify patterns of differential gene expression and gene presence in strains isolated from IPD. Recent studies which used whole-genome sequence data failed to identify adaptive differences, in terms of presence and absence of genes or genetic mutations, between strains invading the blood and strains that were able to cross the blood-brain barrier^11–13^. These works highlight the need of future research to comprehensively identify whether adaptation of IPD isolates occurs through genetic variation between carriage and invasion, suggesting that subtle changes may influence the virulence of the bacterial isolates.

Here, we sought for genetic changes between pneumococcal carriage and IPD isolates using a whole genome sequence typing approach. In this approach, a reference genome is designated, every gene in a data set is assigned an allelic coding based on the reference genome, and new alleles are defined as gene variants containing any change from previously defined alleles (see Methods). This is an extension of the well-known multilocus sequence typing (MLST) method^14,15^. However, in contrast to MLST, which is based on a small number of conserved genes usually present in all isolates of a bacterial species, sequence typing of an entire bacterial genome may contain substantial variations in genes presence.

Hence, using a particular reference genome for such an analysis might preclude identification of genes not found in a certain serotype. To overcome this caveat, we have constructed a pangenome of our IPD samples and used it as a synthetic reference genome. We employed the random forest algorithm (RFA) – a machine learning algorithm commonly applied on genomic data^16^ and previously used by our group identify genes associated with immune selection and lineage structure in pneumococci^17^. Furthermore, to reduce the confounding effect of genes associated with bacterial lineages rather than IPD, we have performed this analysis on three data sets, each time selecting carriage isolated from a different country (UK, USA and Iceland). These carriage isolates were compared against pneumococcal blood isolates originating from heterogeneous geographical locations. The final result consisted of 43 genes ranked in all three datasets among the top most predictive genes for IPD. We characterize these genes, find that many of them are supported in the literature as associated with IPD, and compare our results to presence-absence and tree-based approaches. Finally, we analyze the identified genes’ length and location on the pneumococcal genome relative to capsule-determining loci.

## Results

We obtained 378 invasive pneumococcal isolates causing bacteremia, from different countries, as presented in Table S1. The number of invasive isolates was limited by public availability of WGS samples marked as isolated from blood. A pangenome of 9032 genes was generated from this data set, from which all genes in the soft-accessory genome (defined as genes appearing in at least 15% of samples) were used as a reference genome for the sequence typing process. The sequence typing process was applied three times: on the invasive disease isolates (n = 378) joined with a data set of carriage isolates from the UK (n = 520), USA (n = 622) and Iceland (n = 622). The three datasets were not combined to a single data set for two reasons: First, comparing the results from three different countries constitutes a more conservative approach, increasing the probability of finding genes truly associated with invasive disease, rather than associated with lineages more prevalent in certain datasets. Second, the computational complexity of sequence typing increases non-linearly with the number of genomes. Therefore, we used all carriage isolates available from the UK, and as many isolates from Iceland and the US as possible while maintaining a total of no more than 1000 sequences (the limit in BIGSdb, the web server used for the typing service – see Methods).

Following this rationale, RFA was applied to each of these data sets with invasive/non-invasive disease as the predicted variable. The out-of-bag (OOB) classification success^18^ was similar for the three datasets with 94.6% (95% CI 94.6–94.7), 93.2% (95% CI 93.1–93.2), and 94.4% (95% CI 94.4–94.5) success for carriage, and 76.7% (95% CI 76.7–76.8), 86.7% (95% CI 86.7–86.8), and 79.7% (95% CI 79.6–79.8) success for bacteremia in Iceland, the UK, and USA, respectively. For each data set, the top 100 genes with the highest importance score were chosen, using a heuristic method aiming maximize the number of joint genes (see Methods), and then recorded and compared. Out of these, 43 were joint to all three data sets. The probability of this many, or more, genes joint to all three data sets in a random selection of 100 genes (i.e. the p-value for the null hypothesis of the RFA choosing genes randomly) was verified via simulations to be <10^−6^. Furthermore, RFA was run again using only the 43 genes joint to the three data sets. The OOB classification success was 93.2% (95% CI 93.1–93.2), 96% (95% CI 95.9–96), and 95.9% (95% CI 95.9–96) for carriage, and 73.4% (95% CI 73.3–73.4), 90.1% (95% CI 90–90.2), and 80.6% (95% CI 80.4–80.7) success for bacteremia, in Iceland, the UK, and USA, respectively. The comparable accuracy when using only the 43 joint genes indicates that they are providing sufficient information to classify invasive versus non-invasive pneumococcal strains. All the identified genes are presented in Table 1. We compared our method to two established analysis methods: first, we repeated the analysis based on a genome-wide presence and absence of genes, rather than their alleles, using the software Scoary)^19^ (see Methods for details). No genes were identified as jointly highly predictive in all three data sets using Scoary, even when the top-300 ranked genes were considered. We then applied a sequence-based maximum likelihood phylogeny of the core genes of each dataset^20^. This method also could not capture the evolutionary changes between the invasive and carriage isolates, as these isolates remained scattered across different clades (see Methods and Supplementary Material Figs S3–S5).

**Table 1:** Genes associated with IPD.

Gene	Length (bp)	Best matches, identity (%), e-value, Accession number	Information
*hpp1*	452	1. phtB, 9E-132, 447/465 (96%), NCBI, AF318954.1 2. phpA, 0, 451/480 (94%), NCBI, AF340221.1	1. PHT proteins (aka BHV) are thought to be involved in the invasion process of pneumococci^59,60^. 2. The PhpA protein elicits protective immune response against bacteremia and nasopharyngeal carriage in mice^59^.
*hpp2*	330	hypothetical protein (CPS), 4E-168, 328/330 (99%), NCBI, JQ653094.1	This is a putative capsular polysaccharide biosynthesis protein, Capsular differences are known to be associated with invasive disease^7^.
*hpp3*	249	hypothetical protein	
*hpp4*	504	phtD, 0, 504/504 (100%), NCBI, KP127799.1	The found phtD hit was a part of a sequence shown to be highly conserved in invasive isolates^61^.
*hpp5*	954	Hypothetical protein (CPS), 0, 954/954 (100%), NCBI, HE651314.1	This is a putative capsular polysaccharide biosynthesis protein. Capsular differences are known to be associated with invasive disease^7^.
*hpp6*	996	Hypothetical protein	
*hpp7*	231	pspC, 1E-67, 167/179 (93%), NCBI, AF154043.2	pspC was shown to be involved in immune response to bacteremia in mice^36^.
*hpp8*	510	Hypothetical protein	
*hpp9*	324	Hypothetical protein (CPS), 2E-161, 320/324 (99%), NCBI, ADM91299.1	This is a putative capsular polysaccharide biosynthesis protein. Capsular differences are known to be associated with invasive disease^7^.
*hpp10*	504	pspC,0, 502/504 (99%), NCBI, AF154022.1	pspC was shown to be involved in immune response to bacteremia in mice^36^.
*hpp11*	306	Hypothetical protein	
*hpp12*	399	Hypothetical protein	
*hpp13*	327	Hypothetical protein (CPS), 0, 511/528 (97%), NCBI, AF316639.1	This is a putative capsular polysaccharide biosynthesis protein. Capsular differences are known to be associated with invasive disease^7^.
ydcP_1	471	putative protease YdcP, 0, 470/471(99%), NCBI, AFS43444.1	YdcP is part of the U32 protease family. It is a collagenase, facilitating breaking of extracellular structures tissues, and is a known virulence factor in other bacterial species^62^.
*hpp14*	519	Hypothetical protein (CPS), 0 509/519 (98%), NCBI, AF154022.1	This is a putative capsular polysaccharide biosynthesis protein. Capsular differences are known to be associated with invasive disease^7^.
*hpp15 (hmo)*	147	L-lactate dehydrogenase (FMN-dependent)-like/alpha-hydroxy acid dehydrogenase, 4E-70, 147/147(100%)	Lactate dehydrogenase was found to be essential enzyme for pneumococcal survival in blood^63^.
*hpp16*	480	Hypothetical protein	
*lytB*	1977	Putative endo-beta-N-acetylglucosaminidase, 0, 1968/1977 (99%), NCBI, AJ870414.1	*lytB* codes for a endo-beta-N-acetylglucosaminidase, which is responsible for cell-wall hydrolysis and is thought to be a virulence factor^27,28^.
*hpp17*	528	Hypothetical protein (CPS), 511/528 (97%), NCBI, JF301964.1	This is a putative capsular polysaccharide biosynthesis protein. Capsular differences are known to be associated with invasive disease^7^.
*hpp18*	516	pspC, 508/516 (98%), NCBI, AF154043.2	pspC was shown to be involved in immune response to bacteremia in mice^36^.
*hpp19*	489	Hypothetical protein	
*hpp20*	387	Hypothetical protein (partial transposase), 0, 387/387(100%), NCBI, ADM91518.1	Part of the mobile genetic elements of the bacterium.
*hpp21*	258	Hypothetical protein	
*hpp22*	288	Hypothetical protein	
*hpp23*	210	Hypothetical protein	
*hpp24*	387	Hypothetical protein (partial transposase), 0.0, 386/387(99%), NCBI, CP002176 (positions 1374937–1375323)	Part of the mobile genetic elements of the bacterium.
*hpp25*	510	Hypothetical protein	
*hpp26*	168	Hypothetical protein	
*hpp27*	489	pspC, 0, 463/490 (94%), NCBI, AF154022.1	pspC was shown to be involved in immune response to bacteremia in mice^36^.
lox	1137	Lactate oxidase (lox) gene, 0, 1001/1137(88%), NCBI, DQ984140.3	The *lox* gene is involved in bacterial niche competition and virulence in streptococci and other bacterial species^30,31^.
*hpp28*	840	Sortase (srtA), 0, 614/740 (83%), NCBI, KX147105.1	In *Streptococcus mutans*, disruption of the sortase (srtA) gene led to decrease in adherence and invasion to endothelial cells^64^.
*hpp29*	189	Hypothetical protein	
*hpp30*	537	Hypothetical protein	
*hpp31*	504	Hypothetical protein	
*hpp32*	309	Hypothetical protein	
*hpp33*	309	Hypothetical protein	
cpsA	1446	cpsA (aka wzg), 0, 1446/1446 (100%), NCBI, KC522490.1	wzg (aka cpsA) is part of the capsular polysaccharide synthesis gene locus. High expression of cpsA is associated with bacteremia in humans^65^.
bgaA	6702	bgaA (Beta-galactosidase BoGH2A), 6466/6704 (96%), NCBI, AF282987.1	bgaA is hypothesized to be a pneumococcal virulence factor^66^ and was shown to promote resistance to immune cells in human serum^67^.
cpsA	1446	cpsA (aka wzg), 0, 1446/1446 (100%), NCBI, KC522492.1	wzg (aka cpsA) is part of the capsular polysaccharide synthesis gene locus. High expression of cpsA is associated with bacteremia in humans^65^.
*hpp34*	207	Hypothetical protein	
*hpp35*	573	pspC, 0, 566/573 (99%), NCBI, AF154043.2	pspC was shown to be involved in immune response to bacteremia in mice^36^.
*hpp36*	684	cpsD, 0, 682/684 (99%), NCBI, AFC94091.1	cpsD mutations were shown to inhibit the possibility of causing bacteremia in mice^68^.
*hpp37*	840	Hypothetical protein	

Interestingly, 23 of the genes we identified had BLAST matches with genes previously found to be associated with invasive disease or associated with immune response to it. 18 genes were found to encode for hypothetical proteins with unknown functions and 2 genes were found to encode for transposases, which catalyse the rearrangement of mobile genetic elements in the bacterial chromosome^21^. As a control measure, we performed a similar BLAST analysis on 43 of the jointly lowest-ranked genes (i.e. the worst predictors as determined by the RFA). These genes were comprised of 8 ribosomal genes, 7 metabolism genes, 3 translation/transcription regulation genes, 4 bacteriocin-related genes, and various conserved hypothetical proteins (SI Table S5). The only gene found to be related to virulence was *ilvE*, which is an aminotransferase also relevant for lung infection^4^. As our isolates were derived either from the nasopharynx or from patients’ blood, it might be expected that this gene will not be a highly ranked. Thus, virulence-related elements were over-represented in our top ranked genes, as we would expect from genetic elements associated with invasive pneumococcal isolates.

**Figure 1:**
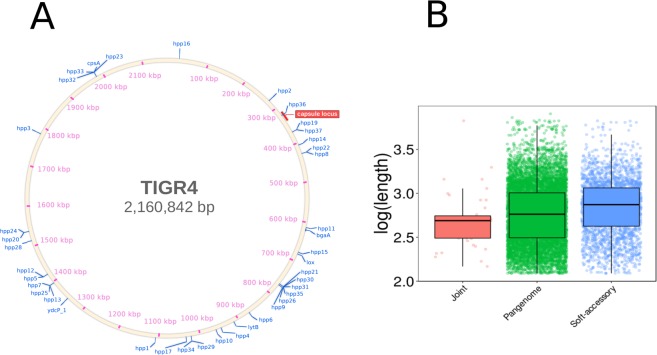
Location and length of genes associated with IPD. (**A**) Location of identified IPD-associated genes (see Table 1) on a 19A streptococcal genome (accession NC_010380.1). Orange rectangle marks the capsular synthesis locus (CPS). Similar plots using other serotype samples can be found in Figs S5–S6. **(B)** Boxplots and distributions of log_10_-transformed gene lengths from the IPD-associated genes, the entire pangenome and the soft-accessory genome used in our analysis (see methods).

We explored the characteristics of the identified IPD-associated genes by determining the locations of the identified genes on the pneumococcal genome. Figure 1A shows the locations of the identified genes across the genome of a 19A serotype sample and reveals that they are spread across the pneumococcal genome. Pneumococcal serotypes are known to be differentially associated with IPD^22,23^, and hence genes located around the capsule polysaccharide synthesis locus might be expected to be involved in IPD. Indeed, several identified genes are found near this gene cluster (orange rectangle on Fig. 1A), but many of the other genes identified are spread across the genome, verifying that our findings do not simply rely on differences in serotype compositions between the datasets used for our analysis (for serotype distribution in our data, see Fig. S1). We note that since pneumococcal serotypes have substantial genomic variation, driven by recombination, horizontal gene transfer and events of gene loss or addition, the locations of genes within the their respective genomes are not constant^24^. Regardless, qualitatively similar location distributions were obtained when plotting these genes on other serotype samples (SI Figs S5, S6). In addition to this, we examined the length of the IPD associated genes (Fig. 1B), since many of them were found to have BLAST matches to short subsets of known genes (see Table 1). The IPD associated genes were statistically significantly shorter than those in the soft-accessory genome (Wilcoxon rank-sum test, p-value = 0.00027) but not significantly shorter than those of the entire pangenome (Wilcoxon rank-sum test, p-value = 0.079). Furthermore, the length of genes in the entire pangenome was significantly shorter than those in the soft-accessory genes (Wilcoxon rank-sum test, p-value = 10^−16^). I.e., the genes we identified are comparable in length to those in the entire pangenome, but are shorter than the soft-accessory genes we used as a reference. Finally, we have examined the variation in the identified genes. In both the invasive and the carriage isolates, the identified genes had more allelic categories than genes not identified by our method (Wilcoxon rank-sum test, p-value < 10^−15^). The presence of the identified genes in the isolates ranged between approximately 30–100%, and was similar between the populations, although slightly lower in the invasive isolates (SI Table S2).

Thus it seems that shorter, more variable genes with varying presence across pneumococci, had a higher probability of being associated with IPD.

## Discussion

In this study we identified pneumococcal genes associated with of IPD using a novel method, comprising a combination of several techniques. First, we encoded WGS data by extending multi-locus sequence typing. This approach enables information to be extracted from gene variants, or alleles, as well as from the presence/absence of genes. Consequently, the sequence typing approach outlined is more sensitive to finding variations within genes without losing information due to the absence of genes.

As a reference genome for the typing scheme, we constructed a genome which included any genes existing in more than 15% of invasive samples, namely the soft-accessory genome. We thus avoided relying on genes present in an arbitrary reference genome for our analysis. This is especially important when typing pneumococcal samples, which have highly variable genomes and can yield a core genome shorter than 50% of an average pneumococcal genome^25,26^. Using a reference genome constructed in such a way has proved beneficial, as all but three of the genes eventually identified as associated with IPD were present in fewer than 95% of isolates, categorizing them in the soft-accessory genome (SI Table S2).

We then used an RFA to score the genes by their marginal contribution to improving classification of invasive disease and carriage. Our method was implemented on three datasets of pneumococcal carriage samples isolated from different countries, and the top-ranked genes were reduced to only those that were jointly top-ranked in all three datasets. Selecting the jointly top-ranked genes imposes a stringent cutoff for the identified genes, and reduces potential bias introduced due to local ancestry or population structure. It resulted in a total of 43 jointly high scoring genes out of 100 top-ranked genes associated with IPD – implying a relatively high replicability of results across datasets. Additionally, we applied a presence/absence method and a sequence-based phylogenetic approach, which yielded no significant results joint to all three data sets.

Reassuringly, many of the genes we identified are parts of known virulence factors, or are associated with invasive pneumococcal disease and especially with bacteremia (see Table 1). For instance, our method identified the gene *lytB* as associated with IPD. The LytB protein is involved in the attachment of *S. pneumoniae* to human nasopharyngeal cells *in vitro*, and its loss was shown to heavily impair the pneumococcal virulence in a mouse sepsis model^27,28^. Additionally, it was shown that these proteins are essential for a successful biofilm production and act to avoid pneumococcal phagocytosis^29^. Another gene identified here is the lactate oxidase *lox*. In other streptococcal species, particularly *S. mutans, S. pyogenes* and *S. oligofermentas*, H_2_O_2_-producing lactate oxidase activity was shown to be used in absence of glucose and for niche competition^30,31^. *S. pneumoniae* is also known to use lactate as an energy source in absence of glucose, converting the lactate molecule to pyruvate with consequent production of H_2_O_2_ ^32^. It was also recently demonstrated that *S. pneumoniae* produces hydrogen peroxide in order to facilitate DNA damage, cell apoptosis and ultimately pathogenesis^33^. Interestingly, homologs of the *pspC* gene appear in four instances amongst the genes we identified (namely *hpp7, hpp10, hpp18 and hpp35*). This could be explained by the characteristic polymorphism of the *pspC* gene: it is known to present high copy-number variation as well as numerous alleles in pneumococcal isolates^34,35^. PspC is a bacterial surface protein (adhesin) essential for colonization of nasal tissue, as well eliciting protection against pneumococcal carriage and bacteremia in a mouse model^36,37^. Moreover, it was found to bind to endothelial blood-brain barrier receptors, facilitating bacterial brain invasion^38^. Although the use of PspC was proposed in a non-capsular vaccine, which could confer protection to invasive disease, its high variability have limited its use as vaccine candidate^39^. This repeated identification of several copies of PspC by our method strengthens the gene’s importance as a factor contributing to IPD.

Furthermore, among the genes identified here were two encoding for transposases. It is known that *S. pneumoniae* is characterized by a high level of genomic plasticity, which allows to the bacterium to react quickly to changes in environmental conditions^40^. As mobile genetic elements are responsible for the dissemination of phenotypic characteristics in the bacterium, such as antimicrobial resistance^41^, and are overexpressed in conditions related to virulence, such as during biofilm production^42^, it is possible to speculate that these mobile genetic elements could be associated with the dissemination of virulence factors amongst the *S. pneumoniae* species.

Most of the other identified genes were hypothetical, with no known function. Based on our method’s classification success, the fact that the highly ranked genes were identified in the analyses of three independent carriage datasets, and the high presence of known virulence factors among the genes, we believe that the hypothetical genes identified are highly likely to be involved in pneumococcal invasive disease. Of particular interest are identified genes which are farther from the capsular locus (see Fig. 1A), which could potentially be serotype-independent IPD-associated genes and therefore relevant across streptococcal strains. The length of the identified genes was also unusually short relative to the synthetic reference genome we used (Fig. 1B), implying that some previously overlooked short gene/protein sequences may also be involved in IPD. Our analysis suggests that further focus should be turned to shorter sequences and gene fragments, which could be factors contributing to IPD. For comparison, we analyzed the 43 jointly lowest-ranked genes, yielding hits in ribosomal, transcription and translation regulation, metabolic and bacteriocin-related genes, together with conserved hypothetical proteins (SI Table S5).

The main limitation of our method is that all alleles are marked as different ‘states’ of a gene and their degree of similarity/difference is not taken into account. Thus, we can identify which genes are associated with differing phenotypes, but subtler methods will be necessary to discern exactly which alleles are responsible for which phenotypic changes. Other methods using WGS data as input may be able to achieve that, but the vast amount of variables needed to encode features of a full sequence do not easily lend themselves to classification methods. A feasible future extension of our method could be adding variables encoding more information about the alleles, such as structural properties of their resulting proteins^43^. However, such an extension will necessitate an efficient way of combining the genetic and protein information as the interactions between genes and their translated protein characteristics will likely have a substantial effect on the results.

Additionally, our method does not explicitly account for the potential confounding effects of the different population structure of pneumococci sampled from various locations (although we have previously shown that RFA is able to distinguish between genes defining lineages to those defining serotypes)^17^. We aimed to reduce this confounding factor by using carriage isolates from three different countries, and invasive isolates from various countries (SI Table S1). The weak effect of population structure in our data is corroborated by the failure of clustering the isolates to invasive/carriage using WGS and sequence type (ST) based trees (SI Figs S2–S4, S10). Furthermore, examining the different sequence types in our data (which are a proxy for pneumococcal lineages)^44^ shows the mixed distribution of these among the datasets (SI Fig. S10) and a similar shared percent of STs between the carriage and invasive data (SI Table 11), determining that population structure cannot account for the differences between invasive and carriage isolates.

However, by restricting the genes we identify to those that are highly ranked in multiple datasets to reduce confounding by population structure, our method trades sensitivity for specificity. Such an approach may miss genes that are less common in certain datasets, but should reduce the probability of identifying genes that are spuriously correlated with IPD due to sampling or population structure. In light of the multiple identified genes with unknown functions, we considered such a conservative approach appropriate and preferred increasing the certainty of our results over identifying more genes with lower confidence.

Finally, using a pangenome based solely on invasive isolates restricts our findings to genes found in at least some of the invasive isolates. Assuming that most of the relevant genes for invasive disease would be present in some invasive isolates is reasonable if the adaptation for invasive disease is more likely to occur by allelic variations in genes present across pneumococcal types, or by pneumococci gaining new genes facilitating adaptation to invasion. It might, however, disrupt identification of genes that are removed from carriage isolates for adaptation to the invasive environment, if such genes exist. Addressing this issue would be possible by creating a larger pangenome, consisting of all available isolates, but would also be more computationally expensive.

The limitations mentioned above can explain why other genes known to be relevant for IPD, such as *pyl, prtA, lytA, lytB, sodA* and *cbiO, piuA*^7,27,45,46^, were not identified by our method.

We believe the method presented here can be applied to a variety of pathogens to identify genes responsible for virulent phenotypes. We foresee our approach being particularly useful when the examined pathogens share only a small core genome, such as *E.coli*^47^ and *C. jejuni*^48^. The goal of our method is to discern with high confidence genes associated with IPD, or any other phenotype, so their function could eventually be experimentally examined. Accordingly, we hope the hypothetical genes identified in this study will be further analyzed and prove to be useful in our understanding of invasive pneumococcal disease.

## Methods

### Pangenome construction and sequence typing

A total of 378 genome sequences of *S. pneumoniae* strains isolated from invasive disease were downloaded from BIGSdb^49^ with geographical origin of isolates and accession numbers available in SI Table S1.

These genomes were used to build an invasive population pangenome using Roary V.3.6.1^50^. Briefly, each draft genome downloaded from BIGSdb was re-annotated with PROKKA V1.12^51^ and the annotation output was fed to Roary for the pangenome construction. Roary parameters were set to minimum blastp identity 90% and MCL inflation value of 1.5.

For the purpose of this analysis, we included in the pangenome the genes present in the soft-accessory genome, i.e. present in >15% of isolates, for a total of 2649 genes. This pangenome was used as the reference genome for sequence typing of three new datasets, containing the invasive sequences together with each one of the three carriage data sets, namely Iceland, the UK or USA. Under the BIGSdb typing scheme, all gene variations in a dataset (defined by any difference between a gene and any previously recognized gene variants) result in new allelic categories. BIGSdb parameter values were set to the webserver defaults: 70% minimum identity for partial matching; 50% minimum alignment for partial matching; BLASTN word size of 20.

Genome sequences were quality controlled before the pan-genome construction by making sure that the total length of each assembly was between 2.0 and 2.3 Mb (the common genome length of completed *S. pneumoniae* genomes as retrieved from https://www.ncbi.nlm.nih.gov/genome/). Moreover, the absence of low-level contamination was ascertained using Kraken v 0.10.5^52^. Briefly, if more than 5% of the total genome assembly sequence was identified as belonging to a different bacterial species, that assembly was removed from further analysis. As shown in Supplementary Material Figs S8 and S9, satisfactory pangenome saturation was reached in terms of core genome and number of new genes added per new genome^53^.

### Scoary, FastTree, sequence type analysis, and genome-location

A pangenome wide association study (Pan-GWAS) was performed using the Scoary V.1.6.16 pipeline^19^. Three new pan-genomes were built using 3 different datasets, each including the 378 genome sequences from the invasive disease strains and genomes from the carriage strains isolated from either Iceland, the UK or USA.

Each of the three pan-genomes (invasive + carriage strains) was then input to Scoary using the invasive/carriage origin of the strain as classifier for the pan-GWAS pipeline.

The genes representing the core genomes of the three invasive + carriage datasets (present in more than 99% of the analysed isolates) were concatenated and aligned with MAFFT V.7.221^54^. The alignment of the core genome was used to reconstruct the maximum likelihood phylogeny of each group of isolates using FastTree V.2.1^20^ under a generalized time-reversible model. Phylogenetic trees for each datasets were then edited and annotated using Evolview V.2^55^. Genome location plots were produced using BRIG V.0.95^56^ with the genome sequence of strains 19A (NC_010380.1), D39 (NC_008533.1) or R6 (NC_003098.1) as references (Figs 1, S5 and S6, respectively).

Pneumococcal sequence typing was carried out according to the PubMLST guidelines, assessing the allelic profiles of 7 housekeeping genes^49^. For each dataset reported in Fig. S10 (USA + Invasive, UK + Invasive and Iceland + Invasive) a neighbour-joining tree was produced using the alignment of the concatenated sequences of the 7 housekeeping genes and the results were visualised using iTol^57^. The phylogenetic trees and the alignments were produced using the BigSDB – iTol tool^49^.

### Random forest analysis

Random forest was implemented in R using the *randomForest* package V.4.6–12^58^. Allele types were turned into numeric variables in the RFA due to computational limitations. To break any biases such enumerations might introduce, we permuted each allele typing and reran the RFA for 200 times on each dataset^17^. The measure used to rank genes was permutation importance (aka Breiman-Cutler importance). Under this method, variable values are permuted for the OOB data of each tree and the resulting classification error is subtracted from the OOB data error without the variable permutation^18^. The average of this difference across all trees is the permutation importance. These importance measures were ranked for all variables and the rankings were averaged across the 200 permutations of RFA applications on each dataset^17^. The fraction of genes joint to the three datasets was compared as a function of the number of top-ranked genes selected. To reduce noise due to small samples of top-ranked genes, both the fraction of genes and the low bound of a 95% binomial confidence interval (with n = number of top-ranked genes and p = fraction of joint genes) were used. In both measures, the maximum fraction corresponded to using 100 top-ranked genes (Fig. S7). Although similar peaks occur when more top-ranked genes were used, we chose 100 as a conservative threshold (i.e. to reduce the number of false positive genes identified).

### Functional annotation of genes associated with IPD

All gene sequences in Tables 1 and S5 were first functionally annotated using the NCBI conserved domain search engine (https://www.ncbi.nlm.nih.gov/Structure/cdd/wrpsb.cgi). Each DNA and translated amino acid sequence was checked for similarity against known genes and protein using nucleotide and protein blast (megablast and blastp algorithms respectively, https://blast.ncbi.nlm.nih.gov). The combined results of the conserved-domains search and blast are described in Tables 1 and S5.

## Supplementary information

Supplementary Information

SI Table S1

SI Table S2

SI Table S3

SI Table S4

SI Table S5

## Data Availability

Accession numbers for pneumococcal sequences used are listed in SI Table S1; the pangenome built from invasive isolates can be found in SI Table S3.
