# Supplementary material for: Identifying genes associated with invasive disease in S. pneumoniae by applying a machine learning approach to whole genome sequence typing data
Source: Sci Rep. 2019 Mar 11;9:4049. doi: 10.1038/s41598-019-40346-7 (PMC6411942; doi:10.1038/s41598-019-40346-7)
Supplement: Supplementary file 1 — Supplementary Information [file 41598_2019_40346_MOESM1_ESM.pdf]

Identifying genes associated with invasive disease in *S. pneumoniae* by applying a machine learning approach to whole genome sequence typing data – supplementary information

Uri Obolski[*, 1, †], Andrea Gori[2, †], José Lourenço[1], Craig Thompson[1], Robin Thompson[1], Neil French[3] Robert Heyderman[2], Sunetra Gupta[1]

[1]University of Oxford, Department of Zoology, Oxford, UK
[2]University College London, Division of infection and immunity, London, UK.
[3] Liverpool School of Tropical Medicine, Liverpool, UK
[†]Equal contribution

*Corresponding author:
Uri Obolski, Email: UriObolski@gmail.com

<u>Figure S1:</u> serotype distribution by dataset. Colors indicate the invasive isolates (green) and carriage isolates from Iceland (red), the UK (teal) and USA (purple). NT- non-type serotypes, UI – unidentified serotypes.

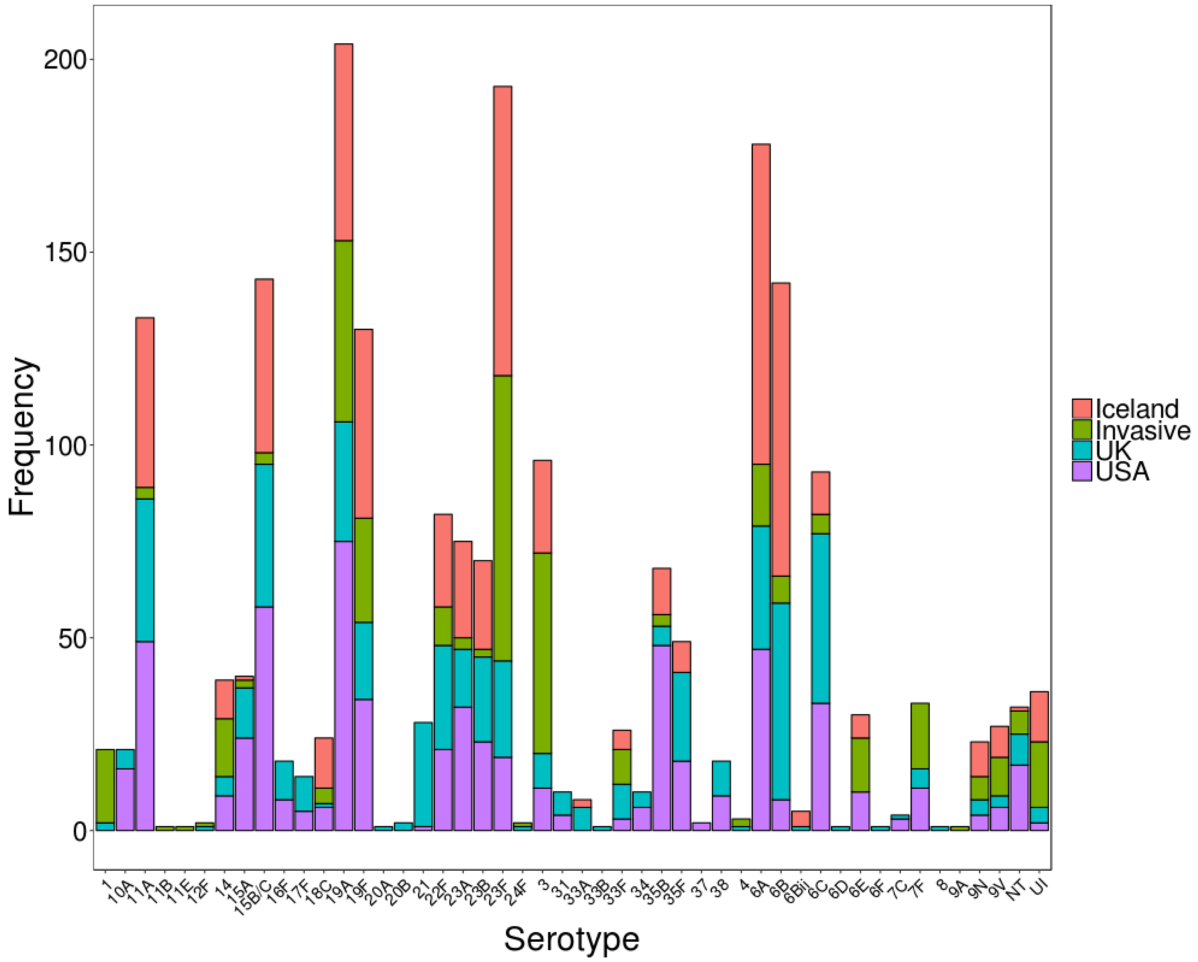

Figure S2: FasTree output for UK.

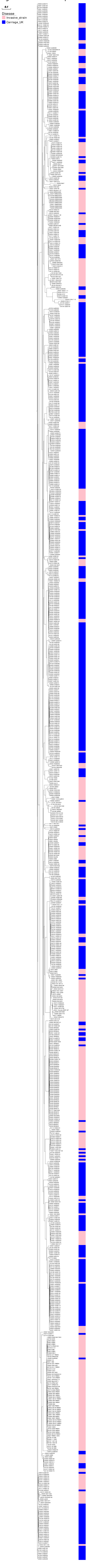

Figure S3: FasTree output for USA.

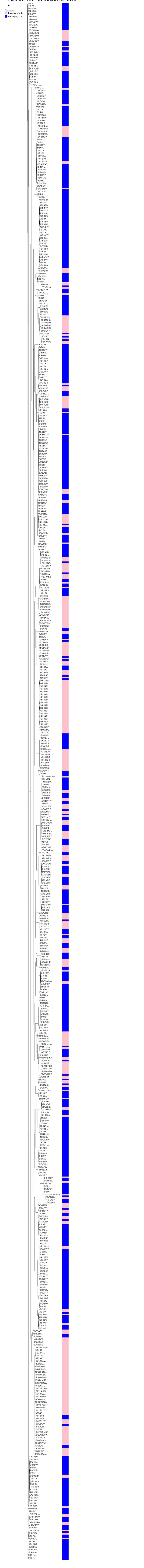

Figure S4: FasTree output for Iceland.

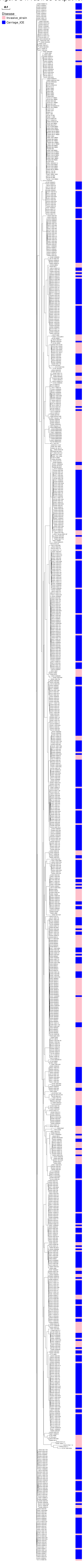

Figure S5: gene location plot for Isolate D39 (accession NC_008533.1).

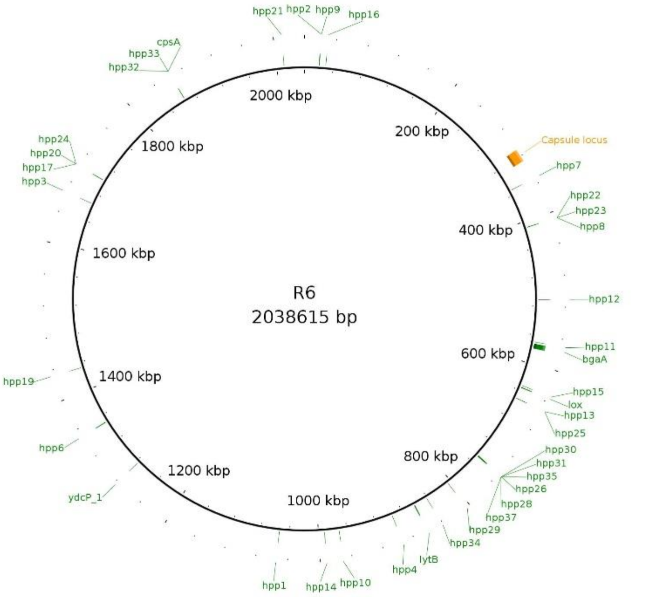

Figure S6: gene location plot for Isolate R6 (accession NC_003098.1).

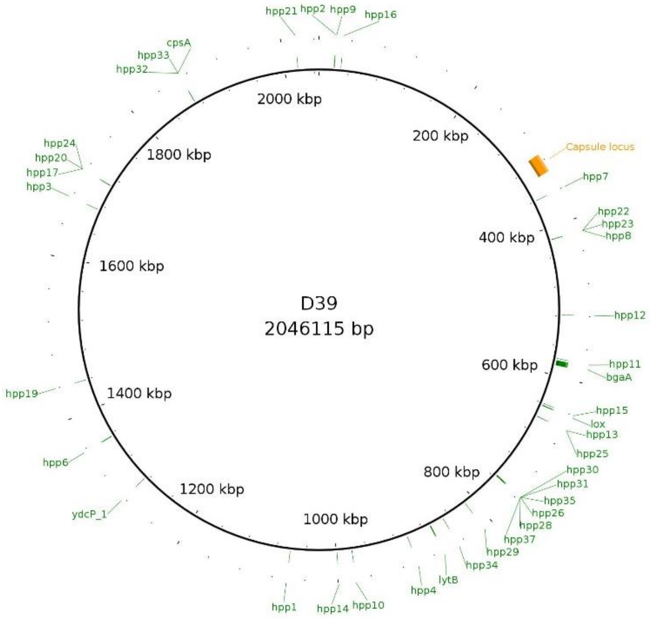

Figure S7: Joint gene fraction as a function of number of top-ranked genes selected. The black curve represents the fraction, while the red is the lower bound of a 95% binomial confidence interval around the fraction. The blue horizontal line indicates the number of top-ranked genes corresponding to the maximum fraction of joint genes (and the low bound of the confidence interval), at 100.

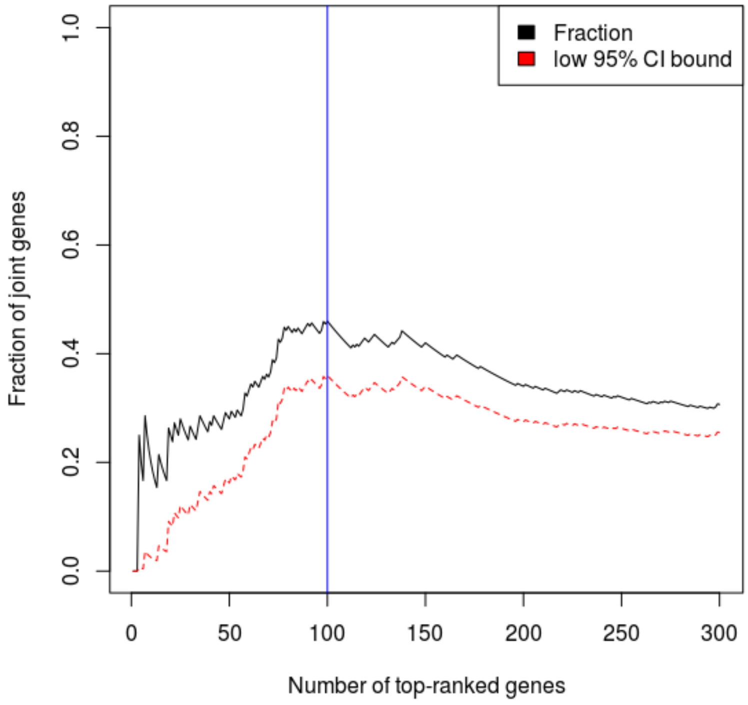

Figure S8: Genes added to the pangenome per new isolate. Boxplots represent the distribution of the number of new genes added to the pangenome for each additional isolate, based on 10 random samples of new isolates.

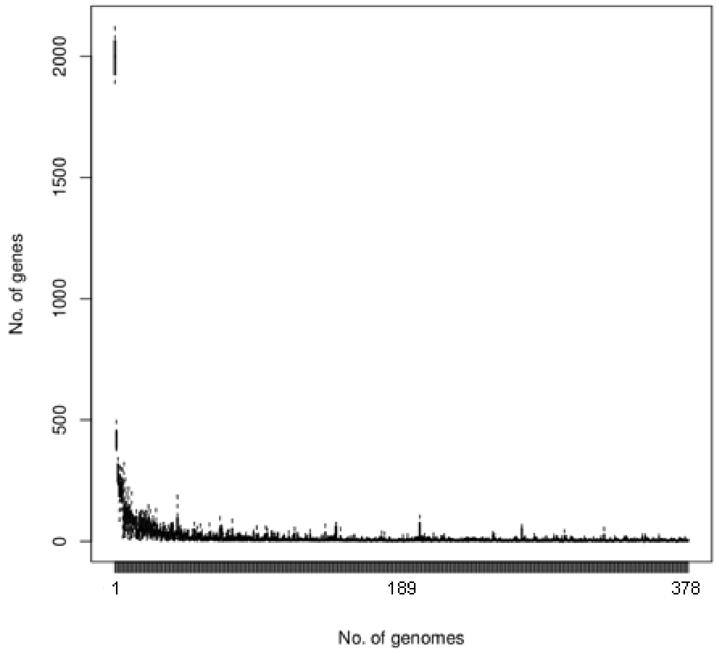

Figure S9: Number of core genes (present in more >99% of strains, dashed curve) and total genes per new genome (solid curve) in the invasive disease pangenome.

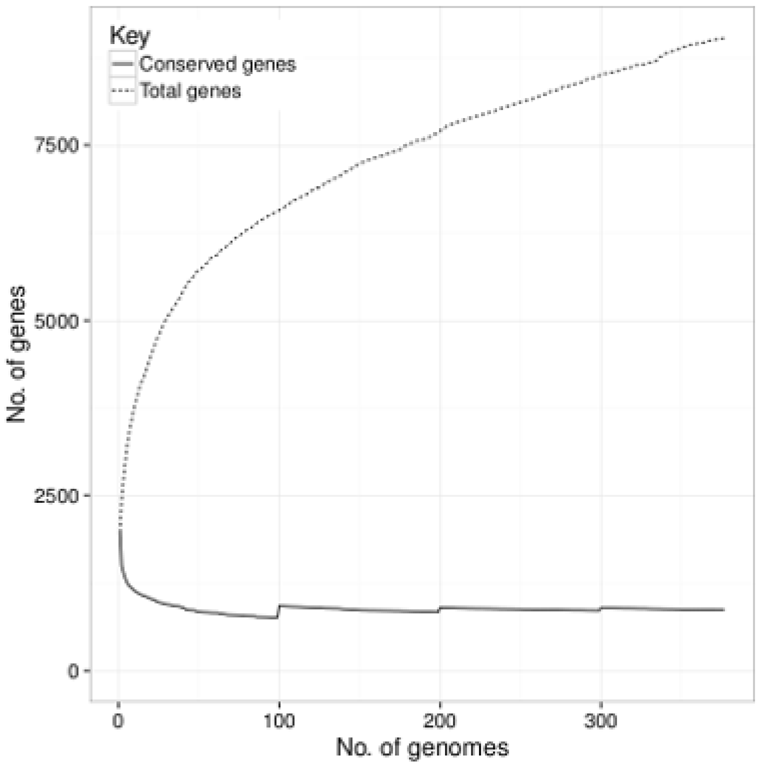

Figure S10: Phylogenetic trees based on STs, colored by country and invasive/carriage isolates.

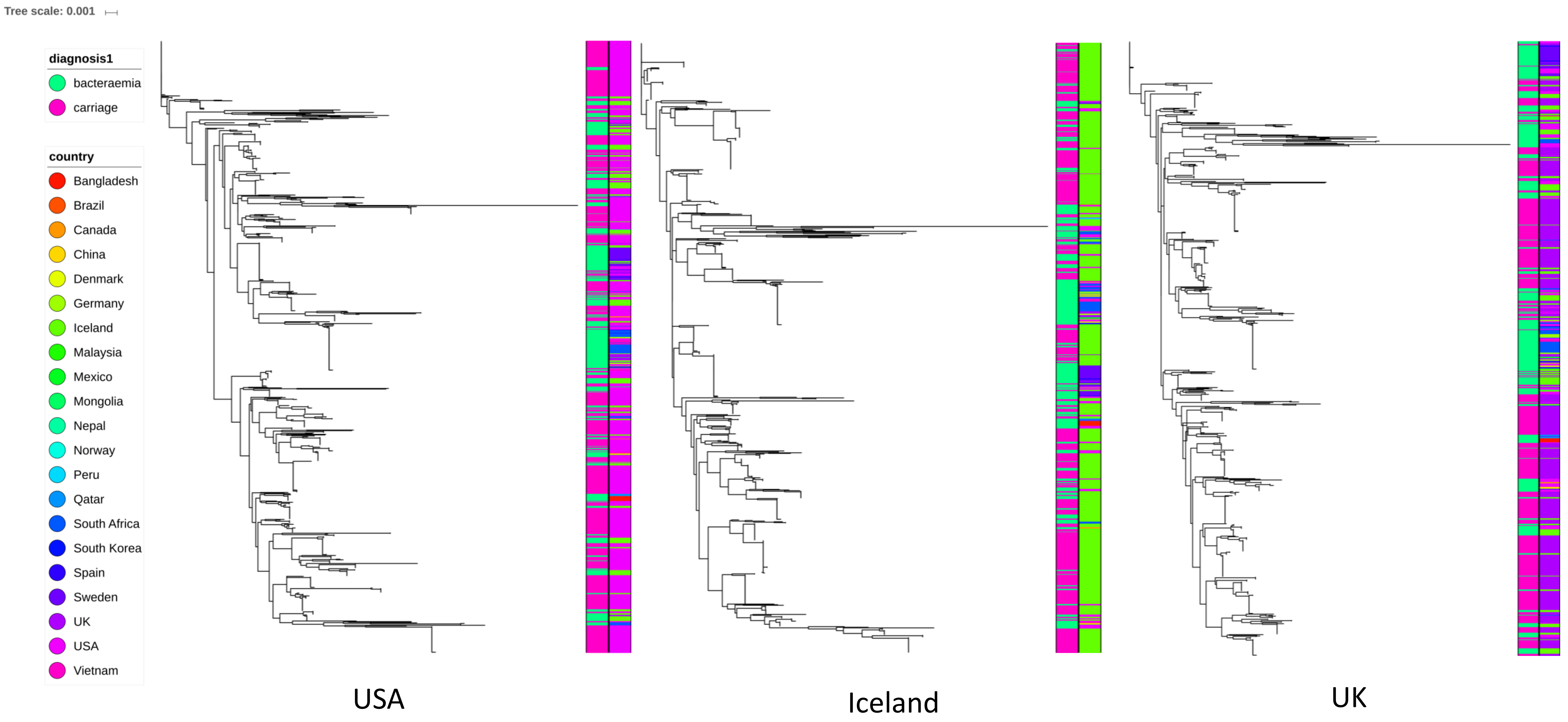

Tree scale: 0.001
diagnosis1
bacteraemia
carriage
country
Bangladesh
Brazil
Canada
China
Denmark
Germany
Iceland
Malaysia
Mexico
Mongolia
Nepal
Norway
Peru
Qatar
South Africa
South Korea
Spain
Sweden
UK
USA
Vietnam
USA
Iceland
UK

Table S1 (attached separately): Pneumococcal sample metadata and accession numbers. Columns, from left to right, indicate the dataset from which isolate was obtained (Invasive disease, USA, UK, or Iceland), the ID number for each isolate's genome assembly as reported on https://pubmlst.org/spneumoniae/, country of isolation, year of isolation, serotype, diagnosis of the individual carrying the isolate (bacteremia or carriage), and the ENA accession number for each isolate's genome assembly (https://www.ebi.ac.uk/ena, where available).

Table S2 (attached separately): Presence of identified genes in isolates.

Table S3 (attached separately): Sequences of identified genes corresponding to Table1 in the main text.

Table S4 (attached separately): Shared STs between populations.

Table S5 (attached separately): Joint 43 lowest-ranked genes.
